# The human gut microbiome in enteric infections: from association to translation

**DOI:** 10.1080/19490976.2026.2612836

**Published:** 2026-01-11

**Authors:** Qi Yin, Samriddhi Gupta, Efrat Muller, Alexandre Almeida

**Affiliations:** a Department of Veterinary Medicine, University of Cambridge, United Kingdom; b School of Public Health and Management, Chongqing Medical University, People's Republic of China

**Keywords:** Enteric infection, pathogens, microbiome, colonization resistance, antibiotics, horizontal gene transfer

## Abstract

Enteric infections remain a leading global cause of morbidity, mortality and economic loss, increasingly compounded by the rise of antimicrobial resistance. The gut microbiome — spanning bacteria, archaea, fungi, protists and viruses — is now recognized as an important mediator that shapes susceptibility to infection, pathogen expansion and disease severity through mechanisms such as colonization resistance, resource competition and immune modulation. Conversely, the gut microbial community can facilitate enteric infection through other processes such as cross-feeding and horizontal gene transfer. In this review, we synthesize correlative and mechanistic evidence currently available on microbiome-pathogen interactions; outline host, environmental and socioeconomic modifiers that affect disease risk across the life course; and evaluate current clinical applications. We highlight key limitations in the field and identify priority areas for future research to refine causal models of microbiome-pathogen ecology and enable targeted diagnostics and therapeutics for preventing and managing enteric infections.

## 1. Introduction

Enteric infections are among the most common form of infectious diseases worldwide.^1^ In 2021 alone, 4.45 billion incident cases of enteric infections were reported globally, with diarrheal disease being the leading cause of death, especially among children under five.^2^^,^^3^ Intestinal infections can be caused by diverse pathogens found across all domains of life. The predominant bacterial pathogens associated with intestinal infections belong to the Enterobacteriaceae family but can include additional species such as *Clostridioides difficile* and *Campylobacter jejuni.*^4^ Beyond bacteria, viruses such as norovirus, rotavirus, adenovirus, and astrovirus, together with protozoa and helminths, similarly represent significant causes of intestinal disease.^5^^,^^6^ There is also a substantial economic impact from enteric infections worldwide. In the United States alone, foodborne illnesses incurred an estimated $17.6 billion in costs in 2018, while in the UK the Food Standards Agency estimates an annual burden of approximately £ 9 billion, with norovirus imposing the greatest economic toll.^7^ Furthermore, because enteric diseases spread through multiple routes (e.g., waterborne, person-to-person and from the environment) they also impose a substantial economic burden in low- and middle-income countries, particularly among young children.^3^ Therefore, there is a crucial need to implement effective mitigation and treatment strategies to reduce the incidence of enteric infections worldwide.

For bacterial infections, antibiotics remain the most common treatment. However, their widespread and often inappropriate use has accelerated the emergence of antimicrobial resistant (AMR) bacteria.^8^ This is especially problematic in low- and middle-income countries, where lack of proper knowledge, misinterpretation of symptoms and self-medication leads to unnecessary use of antibiotics.^8^ Without effective interventions, AMR is projected to cause an additional 39 million deaths worldwide between 2025 and 2050.^9^ To address this global threat, the World Health Organization (WHO) has identified several “critical priority” pathogens for the development of new targeted treatments.^10^ These include carbapenem-resistant *Acinetobacter baumannii*, carbapenem-resistant Enterobacterales, and third-generation cephalosporin-resistant Enterobacterales. The rise of AMR has also prompted increased efforts to search for alternative treatment strategies, including microbiome-based therapies. The human gastrointestinal tract harbors a dense and diverse microbial community — comprising bacteria, viruses, fungi, archaea, and protozoa — that plays a pivotal role in human health.^11^ This diverse gut microbiome supports normal gut physiology, digestion, immune system maturation, and, crucially, colonization resistance against foreign or opportunistic pathogens.^12^^,^^13^ Indeed, many potential enteric pathogens, such as members of the Enterobacteriaceae family, are also common commensals within the gut.^14^ Disruption of gut microbial homeostasis (often referred to as ‘dysbiosis’) is associated with numerous pathologies, including increased susceptibility to pathogen proliferation and enteric infections.^15^ For example, *C. difficile* infections represent a particularly severe and recurrent form of enteric infections linked to antibiotic-induced disruption of the commensal microbiome.^16^ Consequently, fecal microbiome transplantation (FMT) to restore the commensal microbiome of infected patients has been shown to be an effective treatment for *C. difficile* diarrheal disease.^17^

Over the past decades, methodological developments have improved the study of the human gut microbiome and our understanding of its interactions with enteric pathogens.^11^^,^^18^^,^^19^ Culture-based methods have long enabled the isolation of individual bacteria to explore microbial mechanisms individually or in mixed cultures.^20-22^ However, these approaches are limited by the inability to culture many gut microbes — particularly obligate anaerobes — and by biases introduced through selective growth conditions.^23-26^ The advent of DNA sequencing approaches such as 16S rRNA genotyping and whole-genome metagenomic sequencing dramatically improved our ability to characterize the gut microbiome and find associations with pathogen colonization.^19^^,^^27^^,^^28^ Nonetheless, sequencing-based methods also carry constraints, including higher costs, challenges in interpreting complex multi-kingdom datasets, and potential biases introduced during amplification and library preparation.^27^ Thus, experimental approaches to test ecological predictions through *in vitro* and *in vivo* models are still essential and continue to reveal key mechanisms of microbiome-pathogen interactions.^29^^,^^30^

In this review, we examine the current knowledge on the ecological dynamics and interactions between the gut microbiome and enteric pathogens. We focus on known signatures of microbial diversity and composition, positive and negative interspecies interactions, as well as mechanisms of microbiome-mediated colonization resistance and host immune modulation. Finally, we discuss the future implications of these findings and how insights into the gut microbiome ecosystem can be leveraged to develop innovative therapeutic strategies for preventing and managing enteric infections.

## 2. Microbiome associations in health and infection

The human gut harbors a complex community of trillions of microorganisms — approximately 10^13^ bacterial cells — representing thousands of species that co–evolved with their host.^31^ This complex ecosystem protects against enteric pathogens through a combination of direct mechanisms, including niche exclusion, nutrient competition, and production of antimicrobial secondary metabolites; and indirect mechanisms, such as host or environmental modulation.^32^ Therefore, a general imbalance to the gut microbiome, often termed ‘dysbiosis’, is associated with increased host susceptibility to infection. However, the exact mechanisms involved in disease etiology vary according to the baseline microbiome composition of the host and the specific pathogen involved.

Analytical frameworks combining statistical approaches such as differential abundance, network analysis and metabolic modeling applied to large-scale sequencing data have provided important insights into microbial signatures linked to pathogen colonization (Table 1). These DNA-based approaches offer greater sensitivity to detect and profile both the pathogen and the surrounding microbiome composition compared to traditional culture-based methods.^23^ Understanding how the gut ecosystem behaves and changes in the presence or absence of specific enteric pathogens can help further generate hypotheses to be experimentally tested and gain new mechanistic insights. However, the interpretability of microbiome data is largely determined by the original study design. Most microbiome studies rely on cross-sectional, case-control cohorts in which samples are analyzed at a single time point, limiting the ability to distinguish causes from consequences of pathogen colonization.^33^ Although studies of bacterial pathogens have begun to distinguish gut microbial features that predispose individuals to infection^34^ from those that arise because of pathogen colonization,^19^ comparable data for viral and parasitic pathogens remain scarce. This gap reflects several methodological challenges, including the lower abundance of many enteric viruses and parasites, and the need for specialized DNA extraction techniques.^35^^,^^36^ In addition, unlike bacteria, viruses also lack universal molecular markers analogous to the 16S rRNA gene,^37^ making comprehensive profiling more difficult when species are found at a low abundance.

**Table 1.** Examples of microbiome correlations found with enteric pathogens**.**

Species/Pathotype	Disease/Symptoms	Pathogen type	Negative correlation	Reference	Positive correlation	Reference
**Bacteria**						
EPEC (*E. coli*)	Infantile diarrhea Attaching and effacing lesions	Primary, Invasive	*Faecalibacterium prausnitzii* ^a^	[19,28]	*Klebsiella pneumoniae* ^a^	[15,19,28]
			*Faecalibacterium* sp.900539885^a^	[19]	*Intestinibacter bartlettii* ^a^	[19]
			*Faecalibacterium duncaniae* ^a^	[19]	*Intestinibactera* sp.900540355^a^	[19]
			*Eisenbergiella* sp.900066775^a^	[19]	Veillonella sp.900757715^a^	[19]
			*Barnesiella interstinihominis* ^a^	[19]	*Veillonella parvula* ^a^	[19]
EHEC (*E. coli* O157:H7)	Haemorrhagic colitis Haemolytic-uremic syndrome (HUS)	Primary, Invasive	*Choladocola s*p.003480725^a^	[19]	*Ruminococcus gnavus* ^a^	[19]
EAEC (*E. coli*)	Persistent diarrhea	Opportunistic	*Bacteroides dorei* ^a^	[28]	*Bifidobacterium breve* ^a^	[28]
AIEC (*E. coli*)	Chronic ileal inflammation, Crohn’s disease	Opportunistic	*Bifidobacterium bifidum* ^a^	[28]	*Bacteroides thetaiotaomicron* ^b^	[38,39]
*Salmonella enterica* serovar Typhi	Typhoid fever	Primary, Invasive	Clostridiales^b^	[40]	*Enterobacter cancerogenus* ^b^	[43]
			Lachnospiraceae^b^	[40]	*Proteus penneri* ^b^	[43]
*Salmonella enterica* serovar Typhimurium	Gastroenteritis, Foodborne diarrhea	Primary, Invasive	*Bifidobacterium* ^b^	[41]	*Escherichia fergusonii* ^b^	[43]
			*Lactobacillus* ^b^	[41]	*Citrobacter* ^b^	[41]
			*Clostridium spp.* ^b^	[41]	*Prevotella* ^b^	[44]
			*Ruminococcus* ^b^	[41]		
			*Clostridium coccoides* ^b^	[42]		
			*Eubacterium rectale* ^b^	[42]		
*Clostridioides difficile*	Antibiotic-associated colitis Pseudomembranous colitis	Opportunistic	*Clostridium scindens* ^ab^	[45]	*Enterococcus avium* ^ab^	[45]
			*Clostridium populeti* ^ab^	[45]	*Streptococcus thermophilus* ^a^	[45]
			*Blautia hansenii* ^b^	[45]	*Klebsiella pneumoniae* ^a^	[45,46]
			*Bifidobacterium longum* ^a^	[46]	*Enterococcus faecalis* ^ab^	[45,51]
			*Faecalibacterium prausnitzii* ^a^	[47,48]	*Klebsiella oxytoca* ^b^	[45]
			*Roseburia intestinalis* ^a^	[47,48]	*Ruminococcus gnavus* ^a^	[46]
			*Butyrivibrio crossotus* ^a^	[47,48]	*Clostridium perfringens* ^a^	[52]
			*Eubacterium rectale* ^a^	[47,48]		
			*Muribaculum intestinale* ^a^	[49]		
			*Anaerostipes butyraticus* ^a^	[49]		
			*Akkermansia muciniphila* ^ab^	[45,49]		
			*Anaeroplasma abactoclasticum* ^a^	[49]		
			*Clostridium boltea* ^b^	[50]		
			*Blautia producta* ^b^	[50]		
**Viruses**						
Rotavirus	Diarrhea Food intolerance Necrotizing enterocolitis	Primary	*Faecalibacterium*^a^ Ruminococcaceae^a^ Lachnospiraceae^a^	[53-55]	*Escherichia coli* ^a^ *Enterobacter* ^a^ *Klebsiella* ^a^ *Enterococcus* ^a^ *Streptococcus* ^a^	[53,54,56]
Norovirus	Acute diarrhea, Guillain-Barré syndrome (rare)	Primary	*Faecalibacterium*^a^ *Ruminococcus* spp.^a^	[57]	*Enterobacter cloacae* ^a^ *Pseudomonas* ^a^	[58,59]
**Parasites**						
*Giardia lamblia*	Giardiasis, diarrhea, Malabsorption syndrome	Opportunistic	*Bacteroides*^a^ *Alistipes*^a^ *Escherichia*^a^ *Lactobacillus*^a^ *Bifidobacterium* ^a^	[60]	*Prevotella* spp.^a^ *Ruminococcus*^a^ *Campylobacter*^a^ *Enterococcus* *faecium^a^* *Bifidobacterium dentium*^a^	[1,60]
*Entamoeba histolytica*	Amebiasis, Amebic dysentery, Amebic colitis	Primary, Invasive	*Bacteroides* ^a^ *Clostridium coccoides* ^a^ *Clostridium leptu* ^a^ *Lactobacillus* ^a^	[61,62]	*Bifidobacterium*^a^ Lachnospiraceae^a^ Ruminococcaceae^a^ *Alistipes*^a^ *Butyricimonas*^a^	[61,62]
Helminths	Helminthiasis Ascariasis Hookworm infection Schistosomiasis	Primary, Invasive	Bacteroidetes^b^	[63]	Lactobacillales^^b^^ *Mucispirillu*^^b^^	[63]

a Human study.

b Animal models.

Based on the current state-of-the-art, we highlight below some examples of correlative studies that have linked the gut microbiome composition with pathogen colonization and infection.

### 2.1. Bacterial pathogens

Bacterial pathogens can be broadly divided into two categories: (i) opportunistic pathobionts, corresponding to resident gut microbes capable of causing disease under dysbiosis or immune suppression^64^; and (ii) exogenous invasive pathogens, introduced via the fecal-oral route through contaminated water or food, and that actively disrupt intestinal homeostasis.^40^^,^^65^

Members of the family Enterobacteriaceae are prominent examples of both types.^14^ The Enterobacteriaceae family includes known gut commensals^18^ (e.g., nonpathogenic *E. coli* and *K. pneumoniae* strains), opportunistic pathobionts (e.g. adherent-invasive *E. coli* [AIEC]),^66^ as well as exogenous pathogens such as *Salmonella enterica.*^40^ Many of these species are considered high-priority pathogens, as they are implicated in severe infections and an increased rate of antimicrobial resistance.^10^ An overgrowth of Enterobacteriaceae in the gut microbiome is also consistently associated with various negative health outcomes, including chronic diseases such as Crohn’s disease^67^ and an increased all-cause mortality.^68^ Previous work has shown that gut microbiome composition and diversity is strongly linked to Enterobacteriaceae colonization, with specific gut species associated with either co-colonization or co-exclusion.^15^^,^^19^^,^^28^ In general, Enterobacteriaceae colonization has been found to be accompanied by the co-colonization of species from genera such as *Intestinibacter*, *Veillonella*, and *Enterococcus*, while species from the Lachnospiraceae and Ruminococcaceae families are often found to be co-excluders (that is, negatively associated with Enterobacteriaceae). *Faecalibacterium prausnitzii* in particular, a key short-chain fatty acid (SCFA) producer, was inferred the top co-excluder in a recent meta-analysis.^19^ The production of SCFAs results from the fermentation of dietary fibers by gut commensals and are, hence, generally linked to gut homeostasis.^69^ Furthermore, a study examining the ecological dynamics of carbapenemase-producing Enterobacteriaceae (CPE) longitudinally within a year found that decolonization (i.e., the spontaneous loss of CPE from the gut) was associated with higher abundance of commensals known to reduce gut inflammation, including *Bacteroides dorei*, *F. prausnitzii* and *Bifidobacterium.*^28^

In addition to Enterobacteriaceae, several opportunistic pathobionts from the phylum Firmicutes — most notably *Clostridioides difficile* and enterococci — are also key pathogens responsible for enteric disease. *C. difficile* infection (CDI) is a well-documented example of a condition that often follows antibiotic-induced depletion of commensals — particularly butyrate-producing Firmicutes. This creates ecological niches that favor *C. difficile* spore germination and mucosal colonization.^16^ However, the precise mechanisms and gut commensal species behind *C. difficile* colonization resistance are still unclear. Previous studies have shown that patients positive for *C. difficile* show a less diverse Firmicutes population overall, but an increased abundance of Lactobacillaceae, Enterobacteriaceae and Enterococcaceae species.^16^ Furthermore, a recently introduced computational approach referred to as a ‘generalized microbe-phenotype triangulation (GMPT) strategy’ was able to identify 11 taxa associated with *C. difficile* colonization status: 10 with a preventive signal (including *Muribaculum intestinale*, *Anaerostipes butyraticus*, *Akkermansia muciniphila*, and *Anaeroplasma abactoclasticum*) and one permissive species belonging to the *Escherichia* genus.^49^ With regards to enterococci, it was shown that a combination of the commensal species *Clostridium boltea* and *Blautia producta* was associated with colonization resistance against vancomycin-resistant enterococci (VRE) in a mouse model.^50^

Altogether, these studies support a general trend where pathogen colonization and overgrowth is typically characterized by reduced *α*-diversity, overgrowth of Proteobacteria, and decreased SCFA biosynthetic capacity. Lower *α*-diversity often reflects disruption of a stable microbial community, which can reduce competitive barriers that normally prevent pathogen expansion.^70^ Furthermore, Proteobacterial blooms are frequently a marker of inflammation, as they thrive in oxygen- and nitrate-rich conditions generated during host inflammatory responses.^71^ At the same time, reduced SCFA production may weaken colonization resistance by impairing epithelial barrier integrity and diminishing host immune responses that suppress pathogen invasion.^69^^,^^72^

### 2.2. Viral infections

Enteric viruses are a major cause of morbidity and mortality^73^ and include common viral pathogens such as adenoviruses, noroviruses, astroviruses, sapoviruses, and rotaviruses. Rotavirus and norovirus are leading causes of diarrheal mortality, with rotavirus alone reported to have been responsible for ~176,000 deaths globally in 2021 despite widespread vaccination.^74^

Even though viral infections can lead to substantial microbiome changes, whether the microbiome itself directly confers colonization resistance against specific viruses is still largely unexplored. Some prior work has shown that viral gastroenteritis is frequently accompanied by microbiome shifts that can impair colonization resistance and increase the risk of secondary bacterial infections. For instance, across three studies, symptomatic rotavirus infection was consistently linked to a less diverse gut microbiome enriched in Proteobacteria and opportunistic/pathogenic taxa such as *Enterobacter, Klebsiella*, *Escherichia coli*, *Enterococcus* and *Streptococcus*, along with depletion of SCFA-producing anaerobes, including *Faecalibacterium*, Ruminococcaceae and Lachnospiraceae.^53^^,^^54^^,^^56^ In contrast, absence of infection, milder disease or better vaccine response was associated with higher microbial diversity and greater abundance of beneficial SCFA producers. In the case of norovirus, studies have shown that viral infection is linked to a shift from a Bacteroidetes- and bifidobacteria-rich gut community toward increased Proteobacteria (notably *Pseudomonas*) and Firmicutes.^55^^,^^75^ Furthermore, a 16S rRNA genotyping study found that higher abundance of *Faecalibacterium* and *Ruminococcus* spp. was associated with lower IgA concentration against both norovirus and rotavirus, suggesting limited prior infections which could indicate lower susceptibility to infection.^57^

One major limitation of these observational studies is that in the case of viral gastroenteritis, microbiome studies do not usually distinguish between virus-induced changes and those caused by diarrhea itself. Indeed, the microbiome shifts observed could simply be a result of the symptoms commonly associated with gastroenteritis and may not be viral specific.

### 2.3. Parasitic infections

Parasitic enteric infections remain widespread in low- and middle-income countries, particularly in Sub–Saharan Africa, Southeast Asia, and Latin America.^76^ Intestinal parasites include unicellular protozoa (*Giardia lamblia*, *Entamoeba histolytica*, *Cryptosporidium* spp., *Plasmodium* spp.) and multicellular helminths (nematodes, cestodes, trematodes). They impair nutrient absorption, damage the brush-border epithelium, shorten microvilli, and disrupt barrier integrity.^77^^,^^78^ Globally, ~1.5 billion people are estimated to be infected with soil-transmitted helminths.^79^

The interplay between enteric infectious parasites and the gut microbiome is likely multifaceted, as different studies have shown inconsistent associations between parasitic infections with the abundance of several gut microbial species. For instance, although a previous meta-analysis described a *Prevotella*-rich, Gammaproteobacteria-depleted signature associated with *G. lamblia* colonization,^60^ another study linked *G. lamblia* infection to a ‘dysbiotic’ microbial profile characterized by the loss of beneficial commensals and enrichment of potential pathogens (e.g., *Campylobacter*).^80^ This contrasting signal may reflect differences in geography, age, diet, co-infections, and study design. Furthermore, because most studies focus on microbiome changes after infection, we still lack a detailed understanding of the microbial features that predispose individuals to parasitic colonization. With regards to *Entamoeba histolytica* colonization, across two studies^61^^,^^62^ *E. histolytica* infection showed a loss of commensals such as *Bacteroides*, *Clostridium coccoides* and *leptum* groups, and *Lactobacillus*, followed by an enrichment of specific taxa: Verma et al.^62^ noted increased levels of *Bifidobacterium* in infected children, while Morton et al.^61^ found *Entamoeba*-positive adults had increased abundances of several Clostridiales families (e.g., Ruminococcaceae, Lachnospiraceae) and Bacteroidales taxa (including *Alistipes* and *Butyricimonas*). Interestingly, several of these taxa are typically regarded as beneficial commensals. Their higher prevalence in *Entamoeba*-positive adults may be related with host modulatory functions or cross-feeding interactions with other gut bacteria. For instance, mucus secretion is increased in *E. histolytica* infection and this in turn can promote *Bifidobacterium* colonization.^62^ In children with *Cryptosporidium* infection, microbial signatures more closely resembled those observed for bacterial and viral infections, with a depletion of commensal Firmicutes together with a general enrichment of Proteobacteria.^81^^,^^82^

The study of the relationship between the gut microbiome and parasitic worms (e.g., helminths) is also an emerging area of research. Infection of a murine model with *Trichuris muris*, a gastrointestinal parasite, has been associated with a reduced abundance of Bacteroidetes species, alongside an increase of Lactobacillales members and the genus *Mucispirillum.*^63^ Another study investigating the dynamics of *Schistosoma mansoni* infection in mice discovered that baseline microbiome composition was associated with infection risk.^83^ Microbial signals linked to greater worm and egg burdens included low alpha diversity, expanded Proteobacteria and absence of putative immunomodulatory bacteria (e.g. *Lactobacillus*).^83^ Despite these associations, current challenges in the simultaneous profiling of eukaryotic and prokaryotic organisms within metagenomic datasets continue to limit our ability to characterize parasite-microbiome dynamics at a high resolution across large population cohorts.

## 3. Beyond correlation: current mechanistic insights

Over the past decade, research on the gut microbiome and enteric infections has shifted from descriptive correlations to identifying causal mechanisms. Reported associations between community shifts and pathogen burden are now complemented by gnotobiotic models that examine why and how these shifts influence infection outcomes.

**Figure 1.**
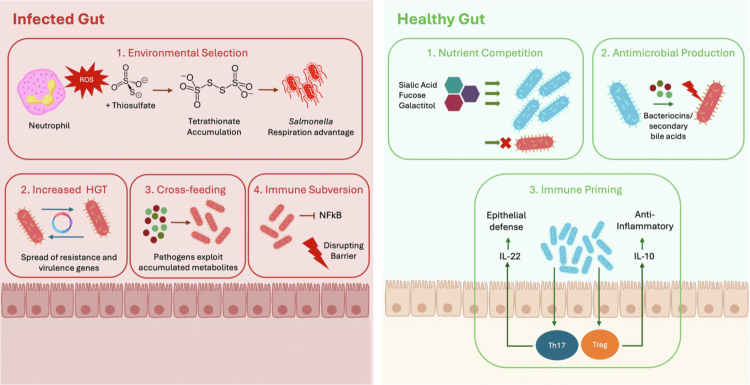
Microbiome-pathogen interactions in infection and health**.** In an infected gut environment (left), disruption of the microbiome favors pathogen expansion through: (1) environmental selection driven by reactive oxygen species; (2) increased horizontal gene transfer that propagates antibiotic resistance and virulence genes; (3) cross-feeding whereby pathogens use accumulated metabolites; and (4) immune subversion via NF-κB-mediated inflammation and disruption of the epithelial barrier. In contrast, in a healthy gut (right), the microbiome limits pathogen colonization through: (1) nutrient competition for host-derived glycans such as sialic acid, fucose, and galactitol; (2) production of antimicrobial compounds including bacteriocins and secondary bile acids; and (3) immune priming that promotes epithelial defenses and anti-inflammatory responses via IL-22, IL-10, and induction of Th17 and Treg cells. This figure has been designed using resources from Flaticon.com.

Within the dense and metabolically active gut, ecological interactions shape whether pathogens are able to establish infection. While commensals and their metabolites can restrict pathogen growth, certain interactions open ecological opportunities for infection (Figure 1). Broadly, these can be divided into positive mechanisms that facilitate pathogen growth, and negative mechanisms that suppress infection.

### 3.1. Positive species interactions

Two of the main mechanisms that critically facilitate pathogen adaptation during infection are horizontal gene transfer (HGT) and metabolic cross-feeding.

#### 3.1.1. Horizontal gene transfer

HGT is the movement of genetic material between coexisting species, facilitated by mechanisms such as conjugation, transformation, and transduction. HGT is a central route through which pathogens rapidly acquire new traits, most notably antibiotic resistance. In a healthy gut, horizontal transfer between commensals and pathogens is thought to be rare due to low pathogen load and a high concentration of metabolites such as SCFAs that inhibit conjugation.^84^^,^^85^ However, infection disrupts this balance. For instance, higher levels of inflammation lead to increased luminal oxygen and suppresses obligate anaerobes.^86^ This in turn promotes Enterobacteriaceae blooms, creating high densities that favor plasmid exchange.^71^^,^^85^ Environmental factors amplify this transfer: a Western-style diet with higher concentration of bile salts in the gut was shown to promote *Salmonella* blooms, increasing plasmid transfer by 10²–10⁶-fold.^87^ Similarly, antibiotics such as *β*-lactams can increase expression of conjugation genes and type IV secretion proteins, leading to higher transfer frequencies.^88^ Additionally, antibiotics may also trigger prophage induction, mobilizing virulence and resistance genes.^89^ Recent mathematical modeling suggests that HGT can even lock microbial communities into pathogen-favouring stable states.^90^

HGT mechanisms exert a significant influence on clinical outcomes. Plasmids disseminate resistance genes such as *blaCTX-M*, *blaOXA-48*, and AmpC *β*-lactamases that drive extended-spectrum and carbapenem resistance in Enterobacteriaceae.^91^^,^^92^ Phages also contribute to the mobilization of virulence factors: prophages encoding the Shiga toxin disseminate *stx* to commensal *E. coli,*^93^ while CTXφ spreads the cholera toxin operon across *Vibrio cholerae* lineages, thereby amplifying infection severity.^94^ Recent work showed that phage-mediated HGT provides stronger contribution to *E. coli* adaptation than mutation,^95^ and probiotic supplementation in preterm infants reduces the prevalence of antibiotic resistance genes and multi-drug resistant pathogens.^96^ Together, these findings highlight that infection-associated HGT is a dominant driver of pathogen adaptation and clinical outcomes.

#### 3.1.2. Microbial cross-feeding

In the healthy gut, cross-feeding interactions stabilize the microbial community, as metabolites produced by one species serve as substrates for another. However, disruption of commensal populations by antibiotics or inflammation can lead to the accumulation of unmetabolized intermediates. For instance, succinate, usually consumed by butyrate producers, can build up and fuel the proliferation of pathogens such as *C. difficile* and Salmonella.^97^^,^^98^ Likewise, sialic acid and fucose released from mucins by *Bacteroides* species are ordinarily taken up by commensals, but in dysbiosis they accumulate and fuel *Salmonella* Typhimurium, enterohaemorrhagic *E. coli* (EHEC), and *C. difficile.*^38^^,^^99^

Inflammatory by-products further exacerbate these effects: nitrate and tetrathionate, for example, enable *Salmonella* to respire ethanolamine and 1,2-propanediol, allowing it to switch from inefficient fermentation to anaerobic respiration.^100^^,^^101^ This gives a fitness advantage to *Salmonella* in the inflamed gut, outcompeting fermenting gut microbes.^100^^,^^101^ Other by-products play similar roles: hydrogen from fermenters supports *Salmonella*^102^ and inflammation liberates proline and hydroxyproline from collagen, which in turn fuel *C. difficile.*^103^ Recent work shows that *Salmonella* can also exploit formate as an electron donor during infection; this requires respiratory pathways activated specifically in inflamed niches.^104^ Furthermore, in *E. coli*, inflammation up-regulates the H47 operon, leading to production of H47 conjugated with the siderophore salmochelin that enables intracellular invasion by *Salmonella.*^105^ Nutritional state also shapes infection outcomes: under protein- and iron- limited malnourished conditions, Bacteroidales liberate mucin- and diet-derived sugars while Enterobacteriaceae increase iron bioavailability, creating a synergistic cross–feeding loop that drives overgrowth of these taxa in both mice and undernourished children.^106^

Host metabolism also contributes to this: when butyrate-producing commensals are lost, colonocytes switch from butyrate oxidation to glycolysis, reducing oxygen consumption and releasing lactate. The result is higher luminal oxygen and lactate levels which fuel Enterobacteriaceae blooms.^86^ Finally, cross-feeding can influence treatment: in a synthetic *E. coli-Salmonella* mutualism model, methionine/carbon exchange on structured surfaces increased persister (non-growing, antibiotic-tolerant subpopulations) formation under ampicillin by up to 55-fold, demonstrating that cross-feeding can not only increase pathogen growth but also antibiotic tolerance.^107^

### 3.2. Negative species interactions

While the above mentioned positive ecological interactions expand pathogen niches, commensals and the host also deploy suppressive mechanisms that limit species growth and virulence. Two major forms of such negative interactions involve the production of antimicrobials and resource competition.

#### 3.2.1. Antimicrobial production

A major process by which gut bacteria inhibit cohabiting or invading species is through antimicrobial production. A recent study used machine learning approaches to identify nearly one million potential new antibiotics in the global microbiome, with 79 of 100 tested peptides showing *in vitro* activity and 63 targeting pathogens.^108^ Enterobacteriaceae in particular produce bacteriocins such as microcins and colicins that use a ‘Trojan-horse’ strategy — they mimic or attach to normal nutrients or transport molecules to sneak past the bacterial cell’s outer defenses and gain entry.^109^ These microcins are largely produced by pathogenic Enterobacteriaceae lineages to gain a competitive ecological advantage over resident commensals.^109^

Commensals also secrete a range of metabolites with antimicrobial properties: lactobacilli secrete bacteriocins, acids, and other metabolites such as the thiol-reactive compound reuterin, which reduces pathogen adherence in epithelial models.^110^ At subinhibitory levels these molecules act synergistically, broadening activity without host damage.^111^ Novel antimicrobials continue to be identified, such as the recently discovered lacriocidin, a lasso peptide that inhibits translation by targeting the ribosome, diverging from typical pore-forming bacteriocins.^112^ Beyond bacteriocins, commensal metabolism generates indirect antimicrobials: for instance, *Clostridium scindens* restores colonization resistance against *C. difficile* by converting primary bile acids into secondary bile acids.^45^ Collectively, these metabolites form a diverse arsenal that restricts pathogen colonization and shapes competitive landscapes during infection.

#### 3.2.2. Resource competition

The nutrient-niche theory, first postulated by Rolf Freter, states that newcomers succeed only by outcompeting residents for limiting resources.^113^ However, when the gut ecosystem is perturbed (e.g., under high inflammatory conditions typical of ‘dysbiosis’), previously occupied nutrient niches become vacant or new substrates are released, creating opportunities for pathogens to expand.^114^ Therefore, resource competition can have multiple ecological outcomes depending on the species composition and environmental conditions. Competitive exclusion occurs when two species relying on the same limiting resource cannot both persist indefinitely, as one species gains advantage and drives the other to local extinction.^115^ In contrast, stable coexistence arises when species differ enough in their resource use, which allows both to be able to persist long-term together.^116^ However, antagonistic interference (e.g., direct suppression between species through antimicrobial production, as described above) can alter outcomes^117^: it may facilitate competitive exclusion if one species suppresses another, or it may promote coexistence if antagonistic effects balance otherwise unequal competitors.

Mechanistic studies support these models: in mice, *E. coli* strains HS and Nissle 1917 have been shown to jointly consume the five mucus-derived sugars required for enterohaemorrhagic *E. coli* (EHEC) colonization, blocking infection only when both strains were present.^118^ This illustrates not only resource blocking but also the possibility of synergistic coexistence, where multiple commensals together generate a barrier no single species can achieve alone. Similar dynamics occur with micronutrients such as iron, where competition can directly suppress pathogen fitness.^119^ In antibiotic-treated mice, commensal *Klebsiella michiganensis* were found to restrict *E. coli* expansion, but this effect was lost when galactitol, a sugar preferentially used by *E. coli*, was supplemented.^29^ Other pathogens are constrained in comparable ways: for instance, *Salmonella* Typhimurium is suppressed when commensals deplete galactitol.^120^ Dietary context can also modulate colonization resistance. *Klebsiella oxytoca* can outcompete *K. pneumoniae* under high-starch or high-sucrose diets,^121^ while dietary L-serine was shown to confer a competitive fitness advantage to Enterobacteriaceae in the gut specifically under inflammatory conditions.^122^

Finally, nutrient competition can have broader consequences: it may restructure community interactions, forcing microbes into new metabolic dependencies such as cross-feeding,^106^^,^^123^ and can also drive pathogen evolution, selecting for variants with enhanced uptake or altered virulence regulation.^124^ Thus, competition for resources within the gut microbiome not only provides immediate colonization resistance but also shapes the long-term ecological and evolutionary trajectories of gut pathogens.

### 3.3. Immune modulation

Commensal microbes modulate three layers of gut defense: epithelial, innate and adaptive to constrain enteric pathogens. We summarize below some examples of each type of interaction in the context of the gut microbiome.

#### 3.3.1. Epithelial layer

Commensal microbes influence gut defense beginning at the epithelial surface. For example, butyrate, a major microbial short-chain fatty acid, plays an important protective role in the gut. It helps defend against *C. difficile* colitis by stabilising epithelial HIF-1 (hypoxia-inducible factor-1), a transcription factor that supports barrier function, and by reducing the production of pro-inflammatory cytokines.^125^ However, the effects of butyrate are not universally beneficial. Recent work shows that butyrate can also limit the differentiation of tuft cells — specialised chemosensory epithelial cells found in mucosal tissues.^126^ This occurs through inhibition of epithelial HDAC3 (histone deacetylase 3). Because tuft cells help initiate type 2 immune responses, especially those required for helminth clearance, this butyrate-driven restriction can dampen type 2 immunity and impair the host’s ability to eliminate parasitic worms.^126^

Pathogens also attack the epithelial barrier directly. *Salmonell*a deploys its effector protein AvrA, which acetylates host MAPKKs (mitogen-activated protein kinase kinases). This modification blocks two major inflammatory signaling pathways: JNK (c-Jun *N*-terminal kinase) and NF-κB (nuclear factor kappa-light-chain-enhancer of activated B cells), thereby dampening host epithelial defense responses.^127^^,^^128^ *C. difficile* uses a different strategy. Its toxins, TcdA and TcdB, glucosylate Rho GTPases, a family of proteins that regulate the cytoskeleton and maintain tight junctions between epithelial cells.^129^ By disabling these molecules, the toxins disrupt epithelial junctional integrity and compromise the barrier.^129^

#### 3.3.2. Innate immunity

Beyond the epithelial interface, commensal-derived metabolites and pathogens both reshape innate immune functions. Butyrate reprograms monocytes into antimicrobial macrophages through HDAC3 inhibition,^130^ increasing early protection against enteric threats. In contrast to commensals, pathogens actively suppress innate immune detection and inflammatory signaling. *Salmonella* provides a well-defined example of this strategy. Its effector protein SopB activates the PI3K-Akt-YAP pathway where PI3K (phosphoinositide 3-kinase) and Akt are central signaling kinases, and YAP (Yes–associated protein) is a transcriptional regulator involved in cell survival and repair.^127^^,^^128^ Activation of this axis inhibits the NLRC4 inflammasome, a cytosolic sensor complex that normally triggers the production of the inflammatory cytokine IL-1β and induces pyroptosis, a form of programmed inflammatory cell death. By blocking NLRC4-driven IL-1β release and limiting pyroptosis, *Salmonella* weakens early innate defenses that would otherwise restrict infection.^127^^,^^128^

#### 3.3.3. Adaptive immunity

Gut bacteria also exert control over adaptive immunity. In mice, segmented filamentous bacteria strongly shape adaptive immunity by promoting the differentiation of two key T-cell subsets: Th17 cells (T helper 17 cells) and Tfh cells (T follicular helper cells). Th17 cells produce the cytokines IL-17 and IL-22, which drive potent antimicrobial responses at mucosal surfaces.^131^ Tfh cells increase the generation of IgA, the dominant antibody isotype in the gut. Together, these SFB-induced pathways improve host resistance to the enteric pathogen *Citrobacter rodentium.*^131^ Defined Clostridia consortia influence a different arm of adaptive immunity. They expand Foxp3⁺ regulatory T cells (Tregs), a population that restrains inflammation and promotes tolerance, through signaling pathways that involve TGF-*β* (transforming growth factor-beta) and IL-10, both of which are immunoregulatory cytokines. Another commensal, *Bacteroides fragilis*, uses its capsular polysaccharide A (PSA) to drive the development of IL-10-secreting T cells. These regulatory cells help protect against inflammatory disease in the gut, including *Helicobacter hepaticus*-induced colitis.^132^^,^^133^ Together, these studies reveal a tug-of-war: commensals and their metabolites program protective immunity, but pathogens evolve counterprograms that redirect these defenses.

### 3.4. Environmental selection

Enteric infections are shaped by environmental selection as well as host- or treatment-driven changes that alter gut physiochemistry and, in turn, influence the gut microbiome. Early studies using metabolic and ecological models derived from human gut metagenomic data revealed that phylogenetically similar species — those predicted to strongly compete for the same resources — tended to co-colonize the human host.^134^^,^^135^ This finding was recently reinforced by a large-scale ecological analysis of Enterobacteriaceae dynamics, which demonstrated that pathogen co-colonization was strongly associated with lower interspecies metabolic distances.^19^ Together, these observations suggest that microbial assembly in the human gut is primarily driven by habitat filtering, an environmental selection process that favors the coexistence of functionally similar species. This mechanism may also help explain apparent discrepancies between *in vivo* studies and observations in human populations. For example, a recent study showed that a defined consortium of 18 commensal strains could decolonize Enterobacteriaceae in a mouse model.^30^ However, many of these same commensal strains were identified among the strongest Enterobacteriaceae co-colonizers in humans.^19^ Ultimately, these findings indicate that interspecies competition in the human gut may be less pronounced than what is observed in current experimental models, which could relate with inherent differences in the intestinal environment and physiology between humans and other animal hosts.

Several studies have addressed more specifically how the intestinal environment affects pathogen success. It was previously discussed that colonization resistance by the microbiome is maintained in an acidic gut environment together with a high production of SCFAs.^72^ In contrast, during colitis innate immune cells produce large amounts of reactive oxygen species as part of the inflammatory response that can enable Enterobacteriaceae species such as *S*. Typhimurium to outgrow fermentative competitors.^101^ Bile acids also have an important role in the gut ecosystem. They eliminate 7-*α*-dehydroxylating bacteria, lowering secondary bile acids that suppress deoxycholic/lithocholic acid production and protect against infection.^45^ Overall, these examples show how diet, pH and inflammation can affect the gut ecosystem and the balance between commensal and pathogen populations in the gut microbiome.

## 4. Host and environmental modifiers

### 4.1. Age and immune state

Age shapes both microbiome assembly and immune development, together determining colonization resistance. Neonates begin with a low diversity microbiome dominated by facultative aerobes, which dampens colonization resistance and enables pathogen expansion.^136^ This is especially evident among infants delivered by cesarean section, which exhibit higher levels of colonization by opportunistic pathogens from the hospital environment.^137^ Additional factors such as preterm delivery, low birth weight and antibiotics increase the risk of late-onset sepsis and necrotizing enterocolitis (NEC).^138^^,^^139^ Protective factors on the other hand include breast milk, which supplies oligosaccharides and immunoglobulins that promote bifidobacteria growth and reduce NEC incidence.^140^ A gnotobiotic mouse model restricting the infant microbial community at the weaning window showed that arrested maturation impairs immune development and increases susceptibility to enteric infection.^141^ Further experimental models have demonstrated that supplementation with Clostridiales bacteria restores colonization resistance in neonatal mice, underscoring their role in controlling pathogen expansion.^136^

Healthy adults have a more diverse microbiome which, under normal conditions, provides colonization resistance via competition for nutrients and niches, antimicrobial production, and SCFAs.^142^ However, this can be hampered by various factors, such as antibiotics. Interestingly, a 2023 population study showed that an antibiotic-associated rise in antimicrobial resistance genes and plasmids persisted much longer in adults, whereas infant effects were shorter but recurrent in high-use settings.^143^ In older adults, immunosenescence and microbiome remodeling can erode colonization resistance and barrier integrity, predisposing an individual to severe viral and bacterial gastroenteritis. Consistent with this, life-long microbiome transfer from young to aged mice preserved epithelial integrity, improved inflammatory profiles and even coordination ability.^144^ Therefore, shifts in microbiome diversity and immune maturity across the lifespan — from the low-diversity neonatal state through the stable, protective adult community to the declining colonization resistance of older age — collectively determine susceptibility to pathogen expansion and enteric disease.

Immunocompromised individuals also exhibit substantial microbiome disruptions. For instance, in HIV patients, depletion of Firmicutes and enrichment of Proteobacteria correlate with reduced CD4⁺ T-cell counts.^145^ In haematopoietic stem cell transplantation, antibiotic prophylaxis and conditioning regimens can lead to an expansion of *Enterococcus* species which are associated with graft-versus-host disease.^146^ Patients receiving solid-organ transplants also show comparable patterns, with *Enterococcus* or Proteobacteria dominance associated with subsequent bacteraemia.^147^ In the case of intensive chemotherapy, treatment can lead to mucositis which facilitate ectopic colonization of the distal gut by oral taxa.^148^ Together, these findings show that colonization resistance is not static but shifts with host age, immune competence, and environmental exposures, explaining heterogeneous susceptibility to enteric infections.

#### 4.2. Geographic and socioeconomic factors

Geography and socioeconomic conditions can shape microbiome composition and enteric infection risk through aspects such as nutrition, sanitation practices and high community antibiotic exposure. Various studies have shown how gut microbiome composition substantially varies with geographic location. For instance, a large-scale analysis of 168,000 16S rRNA gene samples revealed substantial variation in microbiome composition and diversity across different world regions.^149^ In fact, this variation has been described across multiple spatial scales, including substantial differences between continents, such as those observed between North American, African and Asian populations,^150^ but also within continents and countries.^151^^,^^152^ Geographic differences in gut microbiome composition can arise from multiple interacting factors, including population-specific dietary patterns, environmental exposures, cultural practices, lifestyle differences and host genetics. The seminal study by Yatsunenko et al.^150^ on healthy children and adults from the Amazonas of Venezuela, rural Malawi and US metropolitan areas showed substantial variation in bacterial and functional composition between these three regions.^150^ Some of these major shifts included a higher prevalence and abundance of *Prevotella* species among rural populations.^150^ More recently, an ultra-deep sequencing study of Hadza hunter-gatherers identified 124 species vanishing among industrialized populations.^153^

With regards to the impact of socioeconomic factors, studies have shown that low socioeconomic status (SES) is an important risk factor of infection. For instance, a global meta-analysis showed that factors such as limited education, lower income, crowded living conditions, poor access to medical care, or living in highly deprived communities were associated with higher risk of infection.^154^ Furthermore, similar trends were observed in a study conducted in over 400,000 individuals from the UK BioBank, in which the link between low SES and infection was partly attributed to poor lifestyle and chronic comorbidities.^155^

In low-to-middle income countries, the impact of SES is equally pronounced. Research in Bangladesh showed that undernourished children harbour a developmentally immature gut microbiome, quantified by a microbiome-for-age-Z-score.^156^ This immaturity is only partially repaired by standard nutritional therapy and was shown to transmit growth impairment to gnotobiotic mice.^156^^,^^157^ Further studies have identified further clues linked to this pathology: duodenal samples from stunted children in Bangladesh show decompartmentalization, with over-representation of oropharyngeal taxa (e.g., *Streptococcus*, *Rothia*, *Veillonella*), alongside villous blunting, crypt hyperplasia and inflammatory signatures characteristic of environmental enteric dysfunction (EED).^158^ EED is a syndrome characterized by increased inflammation within the small intestine, which weakens colonization resistance and nutrient uptake.^159^ Affected children are at risk of developing more severe enteric infections, prompting earlier and more frequent antibiotics. These treatments deplete protective anaerobes and select for antibiotic-resistant opportunistic pathobionts, making subsequent infections easier to establish.

New analyses of two large, randomized trials in Bangladesh and Kenya show that improved water, sanitation, handwashing and/or nutrition reduce caregiver-reported child antibiotic use by ~10–35% in Bangladesh, though effects were not seen in Kenya, underscoring geographic variability in adherence, transmission dynamics and health system infrastructure.^160^ Another study reported that nearly all Bangladeshi infants receive antibiotics in the first six months, averaging ~11 courses per child per year. At the microbiome level, antibiotics used in rural Bangladeshi infants transiently lower alpha diversity and enrich *Enterococcus* and *Escherichia/Shigella* abundances, with short-lived increases in resistance determinants.^161^ Collectively, these studies highlight the complex association between geography, socioeconomic conditions and antibiotic exposure in modulating early-life gut microbiome development and enteric disease risk.

## 5. Clinical applications

### 5.1. Disease risk prediction

Given the established link between the microbiome taxonomic and functional composition with enteric infections, specific microbial taxa and/or metabolic profiles may potentially hold promise for patient stratification and clinical support in assessing disease risk. This involves both identifying the most sensitive and generalizable microbiome features that predict future infection (or distinguish severe from asymptomatic infections) and prospectively evaluating these predictors while accounting for other known risk factors. Indeed, a recent large-scale, multi-center, prospective study of over 10,000 European adults found that higher abundances of anaerobic butyrate-producing bacteria were linked to a lower risk of subsequent hospitalization due to infections.^34^ As the primary outcome evaluated in this study included various infection types, with only ~20% of infections being labeled as abdominal, future studies should specifically focus on enteric infections and, ideally, stratify infection cases by the specific causative pathogen.

Other studies have yielded mixed results. In a Peruvian birth cohort followed for two years, bacterial richness and the relative abundances of several genera were significant predictors of subsequent all-cause diarrhea, even when adjusting for covariates such as asymptomatic pathogen carriage, antibiotic exposure, and anthropometrics.^162^ In another birth cohort from Malawi, however, no single bacterial genus was associated with future *Shigella* infection.^163^ Similarly, in a longitudinal cohort of international travellers to a region with a high infectious burden, diversity metrics alone were insufficient for predicting future diarrheal episodes.^164^

These findings suggest that while the baseline gut microbiome has potential as an additional infection risk predictor, prediction models are likely to be context-specific (e.g., tailored to specific populations or specific pathogens). This underscores the need for large-scale, prospective studies in which data collection begins prior to disease onset. Such cohorts could directly address questions related with how generalizable microbiome-based risk factors are across ages, geographies and pathogens; and whether they significantly improve infection prediction beyond known demographic and lifestyle risk factors.

### 5.2. Therapeutic interventions

Beyond assessing disease risk, the rise of antibiotic resistance has sparked a general urgency in developing alternative therapeutic applications to mitigate or treat enteric infections. Commercially available probiotics, including *Lactobacillus rhamnosus* GG and *Saccharomyces boulardii*, have shown moderate evidence for preventing antibiotic-associated diarrhea and shortening the duration of diarrhea in children.^165^ Some studies also suggest a possible role in preventing primary or recurrent *C. difficile* infection, although results remain inconsistent.^165^^,^^166^ Surprisingly, the use of probiotics was found to impair and delay microbiome reconstitution following antibiotic perturbations.^167^ It is worth noting that most commercial probiotics belong to *Lactobacillus* and *Bifidobacterium* and do not directly replace key taxa involved in colonization resistance, such as butyrate-producing Clostridiales or bile acid-producing *Clostridium* spp. Prebiotics, such as inulin and fructo-oligosaccharides, represent alternative strategies to selectively stimulate beneficial taxa, but their effects depend on the pre-existing microbiome composition.^168^ Limitations of these approaches include variable clinical outcomes, dependence on host diet and baseline microbiome, and safety concerns in immunocompromised individuals.^169^ Overall, while probiotics and prebiotics offer promising avenues for supporting gut health, their limited ability to restore key protective taxa and their dependence on host-and microbiome-specific factors highlight the need for more targeted, evidence-based therapeutic strategies.

Another avenue currently explored for therapeutic purposes is fecal microbiota transplantation (FMT). FMT is the process of transferring processed stool from a healthy donor into the gastrointestinal tract of a recipient with the aim of restoring a disrupted gut microbiome.^170^ Typically delivered via colonoscopy, nasoenteric tube, enema, or oral capsules, FMT introduces a complex community of commensal microbes and their metabolites that can outcompete or suppress pathogenic organisms, re-establish metabolic function and improve mucosal and immune homeostasis. FMT is a well-established therapy for recurrent *C. difficile* infection (CDI), with cure rates of over 90% reported in both clinical trials and real-world settings.^171^ Its efficacy is thought to derive primarily from restoring disrupted microbial networks, particularly SCFA-producing taxa such as *F. prausnitzii* and members of the Lachnospiraceae and Ruminococcaceae families. However, donor microbiome variability, lack of standardized preparation methods, and incomplete understanding of engraftment dynamics have led to inconsistent outcomes.^172^ A meta-analysis of 316 FMT time-series across 10 disease conditions showed that donor strain colonization or recipient strain displacement did not reliably predict clinical outcomes.^173^ FMT also raises additional concerns, including the risk of pathogen transmission due to imperfect donor screening and uncertainty regarding long-term effects on host immunity and metabolism.^174^ These challenges have led to further efforts to integrate sequencing-guided microbiome profiling for donor-recipient matching and to monitor post-FMT engraftment.^173^ Defined microbial consortia or live biotherapeutic products are under development as safer, standardized alternatives to whole-stool FMT, aiming to preserve efficacy while improving safety and reproducibility.^175^

## 6. Future directions

**Figure 2.**
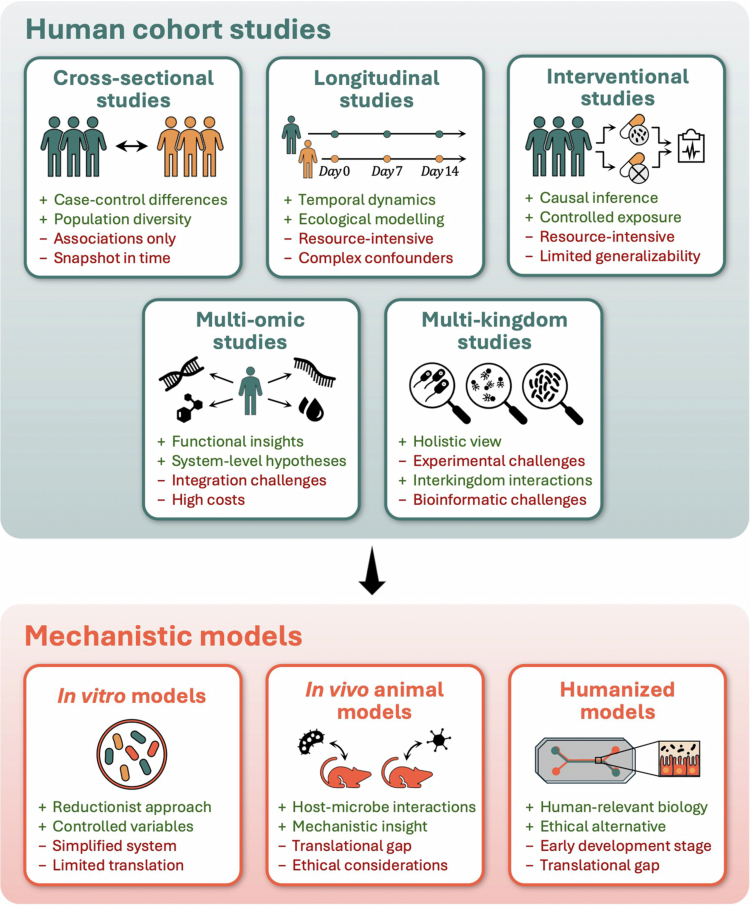
Methodological approaches to study microbiome-pathogen dynamics. Summary of the main advantages and disadvantages of various methodological approaches that can be used to study microbiome-pathogen interactions. Human cohort studies (including cross-sectional, longitudinal and interventional studies) can offer insights into population diversity, temporal dynamics, and system-level patterns but are resource intensive, require large sample sizes and careful consideration of potential confounders. Experimental and mechanistic models enable controlled hypothesis testing and functional insight but vary in translational relevance, complexity, and ethical considerations. This figure has been designed using resources from Flaticon.com.

While previously mentioned studies have established associations and some mechanistic insights between various microbiome features and infection-related phenotypes (e.g., susceptibility to infections, symptom manifestation and severity, long-term morbidities, or recovery following antibiotic treatment), several open questions and technical challenges continue to limit current clinical applications.^176^ Addressing these gaps will require advanced study designs in humans that are specifically aimed at generating causal evidence on microbiome-pathogen interactions beyond correlation. Some examples include longitudinal cohorts and interventional cohorts, including quasi-experimental studies and Randomized Clinical Trials (RCTs). In parallel, multi-dimensional datasets, namely comprising multi-omics and multi-kingdom data, combined with modeling frameworks such as marginal structural models and mediation analysis may be able to support stronger causal inference about microbiome-pathogen relationships. Furthermore, emerging *in vitro* models can complement trial-based evidence by refining mechanistic understanding and bridging remaining knowledge gaps (Figure 2). We expand on each of these points in the following sections.

### 6.1. Improvements to study design

Longitudinal studies, where gut microbiome samples are collected periodically, can characterize temporal dynamics before, during, and after infections, and enable the application of advanced ecological models (such as generalized Lotka-Volterra; gLV). Such models could, for example, provide further evidence of co-colonization or co-exclusion interactions of pathogens with commensals, such as those reported previously in both human and mice studies.^19^^,^^30^ Indeed, a number of studies have successfully used gLV frameworks to infer pairwise interactions and predict temporal trajectories from dense longitudinal microbiome data (e.g., Stein et al. for colonization dynamics in mice intestines;^177^ Clark et al. for synthetic gut communities).^178^ gLV models have also been applied to explore antibiotic-driven dysbiosis and pathogen expansion (e.g., *C. difficile* modeling).^179^ Notably, however, such models have inherent limitations, including challenges with model identifiability and with interpreting compositional, zero-inflated data. Dense sampling over time could also reveal rapid changes in the gut environment that may be missed by conventional pre- and post-infection sampling. For example, in a longitudinal study of European travellers to Laos, daily gut microbiome sampling revealed that all participants acquired extended-spectrum *β*-lactamase (ESBL)-producing Gram-negative bacteria during the first month of their trip, with colonization fluctuating rapidly due to sequential acquisition of multiple strains.^180^

Given the high prevalence and dynamic nature of ESBL-Enterobacterales carriage, and the fact that carriage often precedes infection, future longitudinal studies could help identify microbial or ecological predictors of progression from colonization to infection, or evaluate targeted interventions (e.g., FMT or antimicrobials) to inform antimicrobial stewardship strategies.^180^ Another promising application of longitudinal designs concerns birth cohorts in low-income settings, where rotavirus vaccines show substantially reduced effectiveness compared to other populations.^181^ This reduction may be partly explained by variation in early-life microbiome assembly, vaccine timing, and antibiotic exposure, making longitudinal multi-omics profiling especially valuable for guiding next-generation rotavirus vaccine design.^181^ Such studies could also benefit from an absolute quantification of microbial abundances, rather than relative quantification alone, to account for variation in total microbial load and distinguish between true expansions of specific taxa and apparent increases driven by the loss of others.^182^^,^^183^

Interventional studies in humans represent another important avenue for establishing causal links between microbiome features or microbiome-targeted interventions and infection outcomes. By actively manipulating an exposure such as diet, microbial supplementation, antibiotics, or controlled pathogen or vaccine administration, these studies can directly test mechanisms suggested by observational or multi-omics research. RCTs are especially valuable, and several have evaluated FMT, prebiotics and probiotics for treating or preventing recurrent CDI,^184^^,^^185^ as well as decolonizing drug-resistant pathogens.^186^ Human challenge studies, in which volunteers are deliberately exposed to a pathogen under close monitoring, offer another powerful framework and can be combined with randomized interventions to test microbiome-modulating strategies. Importantly, however, such studies remain rare,^65^^,^^187^ as ethical and safety constraints prevent intentional exposure to most enteric pathogens, and even trials relying on naturally occurring infections require unrealistically large cohorts due to low and unpredictable incidence.

### 6.2. Integrating multi-modal datasets

Multi-omics studies, combining metagenomics, metatranscriptomics and metabolomics, can help identify specific microbial functions, enzymatic activities or metabolites that influence pathogen virulence factors or colonization resistance. In a multi-omics study of *S*. Typhimurium in a mouse model, for example, authors demonstrated that the pathogen was able to withstand various stresses imposed by the host by measuring increased levels of stress response-related proteins with LC–MS proteomics.^188^ In another multi-omics study of *S*. Typhimurium infections, authors integrated metagenomics with metatranscriptomics to identify microbiome members with active metabolic roles during infection, highlighting taxa whose transcriptional activity may promote or restrict *Salmonella* colonization in the inflamed gut.^189^ While these approaches provide rich mechanistic insights, they also have limitations, including cost, technical challenges, and sensitivity to sample collection and storage conditions. Future studies could also perform similar analysis for multiple other pathogens, ideally obtaining omics data from human fecal samples as well as to explore whether findings from mouse models translate effectively to humans. For instance, building on existing findings from *Salmonella* infection studies, additional multi-omics analyses of human fecal samples, integrating metagenomics with host transcriptomics, lipidomics, or metabolomics, could help delineate the metabolic niches and stress-response pathways that facilitate *Salmonella* expansion. Such analyses may also clarify how infection disrupts commensal microbiome members and inform metabolite-targeted therapeutics.^188^

Multi-kingdom microbiome studies that simultaneously profile bacteria, archaea, fungi, protists and viruses may also reveal additional insights into mechanisms of colonization resistance and infection outcomes. Some gut fungi, for example, may restore gut homeostasis following antibiotic treatment, and interact with the innate immune system,^190-192^ while other fungi may disrupt bacterial community structure and facilitate pathogen overgrowth.^193^^,^^194^ As another example, gut viruses, particularly bacteriophages, can modulate bacterial populations in the gut in ways that potentially enhance or diminish colonization resistance,^195^ affecting susceptibility to infections such as *S*. Typhimurium.^196^ Bacteriophages have also been shown to increase pathogen virulence by encoding toxins,^197^^,^^198^ facilitating horizontal gene transfer of various virulence factors through transduction,^199-201^ and transferring antibiotic resistance.^202^^,^^203^ Overall, as accumulating evidence suggests important ecological roles of non-bacterial components in the gut microbiome, multi-kingdom studies represent another promising avenue for future research.

### 6.3. Emerging experimental techniques

While murine models have been invaluable for dissecting gut microbiome-pathogen interactions, important physiological, anatomical and environmental differences between mice and humans mean that mouse findings do not always translate directly. An additional avenue for testing mechanistic hypothesis *in vitro* therefore involves humanized systems that simulate the gut environment, such as human gut organoids or “gut-on-a-chip” devices.^204^ A recent study, for example, used lab-grown human gut organoids to map *Shigella* infection genes, revealing the first genome-wide map of how this human-adapted pathogen invades intestinal tissue.^205^ In another study, human intestinal organoids enabled researchers, for the first time, to propagate noroviruses *in vitro*, representing a breakthrough in noroviruses research and paving the way for diverse future studies.^206^ Still, additional work using human organoid systems is needed to broaden the range of cultivable norovirus strains and improve reproducibility of infection models.^207^^,^^208^ Furthermore, as throughput of such models will increase, so will the potential to perform genome-scale screening of various pathogens, thus resolving molecular infection mechanisms, as recently shown for *Shigella*.^205^Collectively, these emerging methodological approaches promise to transform our understanding of microbiome-pathogen interactions and accelerate the translation of fundamental discoveries into clinical strategies to prevent, diagnose, and treat infection.
